# The Emergence of Turing Instability and Pattern Formation in a Nonlinear Stochastic Spatiotemporal Epidemic Model with Reinfections

**DOI:** 10.1007/s10441-026-09518-7

**Published:** 2026-02-19

**Authors:** Aman Kumar Singh, Rasha Almshekhs, Manish Kumar, Subramanian Ramakrishnan

**Affiliations:** 1 Department of Physics, Maharshi Vishwamitra College, Buxar, BR 802101 India; 2 Department of Mechanical and Aerospace Engineering, University of Dayton, Dayton, OH 45469 USA; 3 Department of Mechanical and Materials Engineering, University of Cincinnati, Cincinnati, OH 45221 USA

**Keywords:** Epidemic modelling, Partial Differential Equations, Turing patterns, Bifurcations, Noise, Stochastic averaging

## Abstract

Instabilities and Turing patterns in stochastic spatiotemporal systems in which a fraction of an evolving population, after undergoing a series of dynamic transitions, returns to its original state, remain largely unexplored. Adopting an epidemic model incorporating reinfections as an exemplar of such a system, we present stability and pattern-formation analyses of the stochastic reaction-diffusion equations that represent the model. Saturation effects in epidemic spread lead to nonlinear considerations, while random environmental effects motivate a stochastic term. Turing bifurcation and the emergence of equilibrium patterns are analysed with respect to three fundamental parameters - reinfection, saturation, and noise intensity. Using higher-order stability analysis and stochastic averaging, we find the Turing instability and also uncover self–organized, distinct equilibrium patterns of infection spread. Additionally, results elucidating the effects of stochastic excitation and its intensity, as well as the competing influence of saturation and reinfection on stability and pattern formation, are presented. The results are also expected to be broadly significant beyond epidemic modelling, for studies of noise-induced instabilities and morphogenesis in spatiotemporal nonlinear dynamical systems.

## Introduction

In 1952, Alan Turing discovered that a homogeneous, *stable* steady state solution of a reaction-diffusion system of partial differential equations (PDE) can become *unstable* owing to the disparity in diffusion coefficients of the two interacting species comprising the system (Turing 1952; Lee et al. 1993; Painter et al. 1999; Meinhardt 1982; Horváth et al. 2009). The universality of the Turing instability earned recognition in the ensuing decades, with the effect being invoked to theoretically explain a broad class of self-organized, pattern formation phenomena, particularly in biological dynamics (Murray 1993; Cass and Bloomfield-Gadêlha 2023)–such as the formation of striped patterns on the skin of a zebra (Caro et al. 2014), on zebra-fish (Kondo et al. 2021), self-organization of cells forming biological patterns (Formosa-Jordan et al. 2023) and so on. Importantly, experimental evidence for the Turing instability was reported in the 1990s (Murray 1993) and has spurred further research ever since (Turing 1952; Dutta et al. 2005; Riaz et al. 2007; Landge et al. 2020). While the Turing instability is engendered by the disparity in diffusion coefficients, it is noteworthy that instabilities corresponding to Hopf bifurcations can also emerge - entirely independent of diffusion - in nonlinear reaction-diffusion systems.

We now turn to the fundamental aspects that motivate our investigation of instabilities and Turing patterns reported in this article. Firstly, despite extensive literature on Turing-type instabilities, instabilities in systems in which a fraction of the population of an evolving species returns to its original state (after undergoing dynamic transitions), remain largely unexplored. In the context of compartmental models describing such transitions, this return of a fraction of the evolving population to the initial compartment may be viewed as closing the loop of the transitions. Indeed, such closure indicates an increase in the population density of the initial compartment, as a fraction of its members returns to it after cycles of dynamic transitions through the other compartments. A pertinent example of such a system studied here is a compartmental dynamic model of an epidemic that accounts for *reinfections*. Here the basic model comprises disjoint compartments (sets) of Susceptible (*S*), Infected (*I*), and Recovered (*R*) individuals, with corresponding population densities denoted by the functions provided within the brackets. The dynamics of an epidemic are described by a system of coupled differential equations in *S*, *I*, and *R*. It follows from the consideration of *spatiotemporal* epidemic dynamics that the model must be formulated in terms of partial differential equations (PDE) since the compartmental densities will be functions of both spatial and temporal variables. While in this framework, individuals typically undergo $S\rightarrow I\rightarrow R$ transitions that characterize an epidemic, reinfections (say, due to loss of immunity or the emergence of new variants of the causative pathogen) will render a fraction of recovered individuals susceptible yet again. Correspondingly, this closes the $S\rightarrow I\rightarrow R$ transition loop, thereby contributing to the dynamic growth of *S* as the Susceptible compartment gains individuals from the Recovered compartment. We note that the onset of instabilities is intimately related to the nuanced dynamic interaction between the species involved; hence the dynamics may be expected to be fundamentally altered owing to the closure of the transition loop. Therefore, understanding the emergence of instabilities and the consequent pattern formation under such dynamics is a prime motivator of the present effort. We focus on the Turing instability in this work (Pearson and Horsthemke 1989; Sun et al. 2007, 2012; Sun 2012; Li et al. 2014).

Secondly, we are motivated to investigate instabilities in the framework of an epidemic model due to the importance of obtaining a deeper understanding of the dynamics of epidemic spread - underscored in good measure as part of the lessons learned from our collective, global experience with the COVID-19 pandemic. In particular, the analysis of instabilities could lead to insights that provide improved analytical characterization of critical drivers of epidemic spread such as superspreader events. In turn, the dynamic analysis will be foundational for control-theoretic analyses that can support and inform rational public health interventions for early and effective mitigation of future epidemics. We note that a detailed discussion and quantitative results that support this line of research were reported in our previous work involving empirical COVID-19 spread data (Majid et al. 2021, 2022).

Thirdly, we are motivated by our recent results in the case of a PDE epidemic model *without* reinfections (Singh et al. 2024, 2023) to study instabilities and self-organized pattern formation in a nonlinear, stochastic, PDE epidemic model wherein reinfections are taken into account. A significant, allied observation is that Turing’s original stability analysis is hinged on *linearized* dynamics around the steady state. While the Turing approach is not directly applicable to nonlinear systems, it has, however, motivated *higher-order* perturbative approaches aimed at uncovering the role of nonlinearity in determining the instability conditions leading to pattern formation in stochastic systems (Riaz et al. 2007; Dutta et al. 2005; Riaz et al. 2005). The study of instabilities in a system with a closed loop of dynamic transitions using a higher-order perturbative approach is yet another novel contribution of the present effort.

An integral aspect of our analysis is the consideration of nonlinearity in the epidemic PDE model. While providing the details later in the article, we now discuss the rationale for considering a nonlinear model. We reiterate that the PDE model is a compartmental model of epidemic spread; a population is partitioned into disjoint subsets of Susceptible (S), Infected (I), and Recovered (R) individuals and an epidemic evolves as the *I* compartment gains individuals due to interaction with the *S* compartment. Mathematically, this transition is characterized by the *infection force*. Supported by empirical evidence, as well as factors such as patterns in human behavior, the infection force is often better represented by nonlinear functions that account for saturation effects (see, for instance, (Capasso and Serio 1978; Xiao and Ruan 2007; Rohith and Devika 2020)). As a consequence the epidemic PDE model becomes nonlinear. Additionally, accounting for external uncertainties that may be fully expected to influence the dynamics of epidemic spread, we consider the PDE for the Infected population density (I) also to be driven by a white noise term. Thus, both nonlinearity and stochasticity play essential roles in our analysis of instabilities in a closed-loop system.

The rest of the article is laid out as follows. The reaction-diffusion type, stochastic PDE epidemic model incorporating a nonlinear infection force characterized by a saturation term is presented in Sect. 2, followed by the derivation of the homogeneous steady-state solution of the model. The higher-order perturbative analysis, followed by the derivation of the evolution equations for the moments (averaged quantities that describe the spread dynamics) is provided in Sect. 3. The stability and pattern formation results are presented in Sect. 4. A discussion of the results and concluding remarks are presented in Sect. 5.

## The Spatiotemporal S–I–R Model

Considering a generic compartmental model as the basis for the analysis, we reiterate that the mathematical model for epidemic spread dynamics comprises three coupled reaction-diffusion type PDEs - one each for the Susceptible (*S*), Infected (*I*), and Recovered (*R*) population densities respectively (Sun 2012; Sun et al. 2016). Reinfections may be characterized as a fraction of the recovered population becoming susceptible yet again; this is mathematically represented by the term $\mu R$ (where $\mu \in [0,1]$) coupled to the PDE for *S*. Turning next to the rate of infection $\beta (I)$, we first note that in basic models without saturation, this quantity is defined as $\beta (I)=\beta _0 I$, where the constant $\beta _0$ represents the per capita contact, and *I* is the infected population density. However, taking saturation effects into account the rate of infection $\beta (I)$ can be modeled as (Xiao and Ruan 2007)

1 $$\begin{aligned} \beta (I)=\frac{\beta _0 I^2}{1+\alpha I^2}, \end{aligned}$$

where the term $1+\alpha I^2$ represents the saturation effect. We note that the saturation effect is well-recognized in epidemiology and readily finds interpretation as arising from changes in human societal behaviour during an active epidemic. Specifically, susceptible populations tend to exercise caution - either voluntarily or owing to restrictions imposed by public health administrations - in their movements and interactions during an epidemic, thereby inhibiting the spread (Gumel et al. 2004). The intensity of the saturation effect (which in turn depends on the intensity of measures such as isolation, quarantine, restriction of public movement, aggressive sanitation, and so on) is captured in the parameter $\alpha$. Indeed, an epidemic spread may also achieve higher levels of saturation owing to voluntary behavioural changes adopted by a susceptible population, including the increased acceptance of protective measures such as social distancing, sanitation, self-isolation, and masking. To summarize, saturation effects that inhibit the spread are represented using the non-monotonic function, $\beta (I)$, given in Eq. (1), where $\alpha$ is a positive constant representing the saturation rate (Xiao and Ruan 2007; Rohith and Devika 2020). However, the rate of infection $\beta (I)=I$ giving rise to nonlinear coupling *SI* have also been analyzed in the literature in the context of the Turing instability (Liu and Jin 2007; Sun et al. 2007).

We now consider the following non-dimensionalized system of coupled PDEs for *S*, *I* and *R* obtained in Eq.(A.2) (Ruan and Wang 2003) together with a noisy perturbation as:

2 $$\begin{aligned} \frac{\partial S}{\partial t}&=b-dS-\frac{\beta _0 S I^2}{1+\alpha I^2}+\mu R+D_1\nabla ^2 S, \end{aligned}$$

3 $$\begin{aligned} \frac{\partial I}{\partial t}&=\frac{\beta _0S I^2}{1+\alpha I^2}-(\gamma +d) I+D_2\nabla ^2I+\xi \left( t,x,y\right) ,\end{aligned}$$

4 $$\begin{aligned} \frac{\partial R}{\partial t}&=\gamma I-(\mu +d) R+D_3\nabla ^2R \end{aligned}$$

constrained by no flux boundary condition, i.e., $\frac{\partial S}{\partial x}=0$, $\frac{\partial S}{\partial y}=0$, $\frac{\partial I}{\partial x}=0$, $\frac{\partial I}{\partial y}=0$, $\frac{\partial R}{\partial x}=0$, and $\frac{\partial R}{\partial y}=0$. The non-dimensional variables *S*, *I* and *R* denote the susceptible (*S*), infected and recovered population densities respectively. Moreover, $\nabla ^2$ is the Laplacian operator representing diffusion. The parameters appearing in Eq. (2) are defined in Table 1.

**Table 1:** The dimensionless parameters of Eq. (2)

Column 1	Column 2
*b*: birth rate of *S*	*d*: date rate
$\beta _0$: transmission rate	$\mu$: re-infection rate
$\gamma$: recovery rate	$D_I$: noise strength
$\alpha$: saturation parameter	$D_1$: diffusion coefficient of *S*
$D_2$: diffusion coefficient of *I*	$D_3$: diffusion coefficient of *R*

$\xi (t, x, y)$ is a spatiotemporal white Gaussian stochastic process with correlation defined as:

5 $$\begin{aligned} & \left\langle \xi \left( t_1,x_1,y_1\right) \xi \left( t_2,x_2,y_2\right) \right\rangle \nonumber \\ & \quad ={2D}_I\delta \left( x_1-x_2\right) \delta \left( y_1-y_2\right) \delta \left( t_1-t_2\right) , \end{aligned}$$

where $D_I$ is the noise intensity.

Let $(S_0, I_0, R_0)$ be the homogeneous steady-state solution of the system described by Eq. (2). The quantities $S_0$, $I_0$, and $R_0$ may be obtained by setting the right-hand side of Eq. (2) to zero, in the absence of both diffusion and noise. Thus $(S_0,I_0,R_0)$ simultaneously satisfy:

6 $$\begin{aligned} \begin{aligned} b-d S-\frac{\beta _0\ SI^2}{1+\alpha I^2}+\mu R&=0,\\ \frac{\beta _0\ SI^2}{1+\alpha I^2}-(\gamma +d)I&=0,\\ \gamma I-(\mu +d)R&=0. \end{aligned} \end{aligned}$$

We note that since the system of equations (6) is nonlinear, $(S_0, I_0, R_0)$ may be more readily obtained as a numerical solution. Moreover, since we seek to understand pattern formation under the influence of reinfections, we do not focus on disease-free dynamics and only consider the endemic case. In the absence of both noise and diffusion, we focus on the stable equilibrium solution $(S_0, I_0, R_0)$ with system parameters chosen accordingly. However, competing populations undergoing diffusion will self-organize through pattern formation. In other words, pattern formation will emerge if diffusion triggers instabilities in the homogeneous steady solution - indeed, this phenomenon lies at the core of the Turing instability. Interestingly, in cases where diffusion alone does not trigger instability, the presence of noise of even a small intensity can induce instability. This remarkable effect is of particular interest in this work.

## Stability and Moments

We reiterate that, compared to our previous work, the novel feature of the model considered here is the reinfection parameter $\mu$. Therefore, of key interest in the analysis is the effect of this parameter, especially in conjunction with the saturation and noise parameters. In order to analyze stability, we now perturb the system from its uniform steady state, i.e., $S_0\rightarrow S_0+\delta S$, $I_0\rightarrow I_0+\delta I$ and $R_0\rightarrow R_0+\delta R$. However, to capture the effects of noise on stability, we find it essential to go beyond standard linear stability analysis and invoke the Taylor series expansion up to the *second order* in the perturbation around $(S_0,I_0,R_0)$. To achieve this we first rewrite Eq. (2) in the following form:

7 $$\begin{aligned} \frac{\partial S\left( x,y,t\right) }{\partial t}&=F(S,I,R)+D_1\nabla ^2 S, \end{aligned}$$

8 $$\begin{aligned} \frac{\partial I\left( x,y,t\right) }{\partial t}&=G(S,I,R)+D_2\nabla ^2I+\xi ,\end{aligned}$$

9 $$\begin{aligned} \frac{\partial R\left( x,y,t\right) }{\partial t}&=H(S,I,R)+D_3\nabla ^2R, \end{aligned}$$

where

10 $$\begin{aligned} F(S,I,R)&=b-d S-\frac{\beta _0\ SI^2}{1+\alpha I^2}+\mu R,\end{aligned}$$

11 $$\begin{aligned} G(S,I,R)&=\frac{\beta _0\ SI^2}{1+\alpha I^2}-(\gamma +d)I,\end{aligned}$$

12 $$\begin{aligned} H(S,I,R)&=\gamma I-(\mu +d)R. \end{aligned}$$

We note that functions *F* and *H* depend on *R* linearly. The corresponding Jacobian matrix in the absence of diffusion for the linearized system, and the diffusivity matrix are given by (Haas and Goldstein 2021)

13 $$\begin{aligned} J=\begin{bmatrix} F_S& F_I& F_R\\ G_S& G_I& G_R\\ H_S& H_I& H_R \end{bmatrix},~~~~D=\begin{bmatrix} D_1& 0& 0\\ 0& D_2& 0\\ 0& 0& D_3 \end{bmatrix}, \end{aligned}$$

where the subscripts in the functions denote partial derivative with respect to the indicated variables. Note that this Jacobian matrix can be obtained from linear stability analysis (i.e. under the first-order Taylor expansion). In the presence of diffusion, the Jacobian matrix (*J*) becomes $J_D(k^2)=J-k^2D$, where *D* is defined in Eq. (13). For the Turing bifurcation, the real eigenvalue of $J_D(k^2)$ crosses zero, i.e., $det(J_D(k^2))=0$. This induces Turing instability first at some $k=k_{th}$ which is determined by the condition $\frac{\partial }{\partial k^2}[det(J_D(k^2))]=0$. This condition yields a cubic polynomial in $k^2_{th}$. In turn, this cubic polynomial will have a double root at ($k=k_{th}$) if the discriminant $\Delta (D_1,D_2,D_3)$ of the polynomial vanishes (Pearson and Horsthemke 1989; Haas and Goldstein 2021). Whilst the aforementioned computations that result in elegant analytic conditions for the onset of the Turing instability are quite straightforward in linear stability analysis, such conditions are arduous to obtain in the case of higher (second) order stability analysis. Furthermore, the presence of noise - the last term on the RHS of Eq. (8) poses additional analytic challenges. Hence, we adopt a different approach based on stochastic averaging, with the objective of obtaining stability results by analyzing the dynamics of averaged quantities (moments) associated with the functions *S*, *I*, and *R*. The averaged dynamics will be described by ordinary differential equations for the moments (sometimes termed moment evolution equations); we now turn to derive these moment evolution equations in the context of our second-order stability analysis.

We begin by substituting the perturbations $S\rightarrow S_0+\delta S$, $I\rightarrow I_0+\delta I$ and $R\rightarrow R_0+\delta R$ in Eq. (7), followed by computing the second order Taylor expansion around $(S_0,I_0,R_0)$ to obtain

14 $$\begin{aligned} & \frac{\partial \delta S}{\partial t}=F_S\delta S+F_I\delta I+F_R\delta R+\left( 1/2\right) F_{SS}\delta S^2\nonumber \\ & \quad\quad\quad +\left( 1/2\right) F_{II}\delta I^2+F_{SI}\delta S\delta I\nonumber \\ & \quad\quad\quad +D_1(\delta S_{xx}+\delta S_{yy}), \end{aligned}$$

where $F_S=\frac{\partial F}{\partial S}$ is evaluated at point $(S_0,I_0, R_0)$. Similarly, we obtain

15 $$\begin{aligned} & \frac{\partial \delta I}{\partial t}=G_S\delta S+G_I\delta I+G_R\delta R+\left( 1/2\right) G_{SS}\delta S^2\nonumber \\ & \quad\quad\quad +\left( 1/2\right) G_{II}\delta I^2+G_{SI}\delta S\delta I\nonumber \\ & \quad\quad\quad +D_2(\delta I_{xx}+\delta I_{yy})+\xi . \end{aligned}$$

16 $$\begin{aligned} & \frac{\partial \delta R}{\partial t}=H_I\delta I+H_R\delta R+D_3(\delta R_{xx}+\delta R_{yy}). \end{aligned}$$

Next we discretize the system of equations Eqs. (14), and (15) at the lattice site (*i*, *j*), to obtain (Riaz et al. 2007)

17 $$\begin{aligned} & \frac{\partial \delta S_{ij}}{\partial t}=F_S\delta S_{ij}+F_I\delta I_{ij}+F_R\delta R_{ij}+\left( 1/2\right) F_{SS}\delta S_{ij}^2\nonumber \\ & \quad\quad\quad +\left( 1/2\right) F_{II}\delta I_{ij}^2+F_{SI}\delta S_{ij}\delta I_{ij}\nonumber \\ & \quad\quad\quad -k^2D_1\delta S_{ij}(t), \end{aligned}$$

18 $$\begin{aligned} & \frac{\partial \delta I_{ij}}{\partial t}=G_S\delta S_{ij}+G_I\delta I_{ij}+G_R\delta R_{ij}+\left( 1/2\right) G_{SS}\delta S_{ij}^2\nonumber \\ & \quad\quad\quad +\left( 1/2\right) G_{II}\delta I_{ij}^2+G_{SI}\delta S_{ij}\delta I_{ij}\nonumber \\ & \quad\quad\quad -k^2D_2\delta I_{ij}+\xi _{ij}(t), \end{aligned}$$

19 $$\begin{aligned} & \frac{\partial \delta R_{ij}}{\partial t}=H_I\delta I_{ij}+H_R\delta R_{ij}-k^2D_3\delta R_{ij}. \end{aligned}$$

where we have used $\delta S(x,y,t)=p(t)\cos {k_x x}\cos {k_y y}$, $\delta I(x,y,t)=q(t)\cos {k_x x}\cos {k_y y}$, and $\delta R(x,y,t)=r(t)\cos {k_x x}\cos {k_y y}$, along with $k^2=k_x^2+k_y^2$. Hence $\nabla ^2\delta S(x,y,t)=-k^2\delta S(x,y,t)$, $\nabla ^2\delta I(x,y,t)=-k^2\delta I(x,y,t)$ and $\nabla ^2\delta R(x,y,t)=-k^2\delta R(x,y,t)$ (please see, for instance (Riaz et al. 2007)). In discrete form, the correlation function of $\xi$ is given by

20 $$\begin{aligned} \langle \xi (t_1)_{ij}\xi (t_2)_{kl}\rangle =2C_I\delta _{ik}\delta _{jl}\delta (t_2-t_1) \end{aligned}$$

where $C_I=\frac{D_I}{\Delta x \Delta y}$ for a grid spacing of $\Delta x$ and $\Delta y$. Discarding the suffixes (*ij*) for notational convenience, henceforth, we shall implicitly assume the reference to the lattice site (*ij*). For example, $\delta S$ stands for $\delta S_{ij}$, and a similar notation is adopted for all other quantities. Next, statistical averaging of Eqs. (17), and (18) yields

21 $$\begin{aligned} & \langle \delta S\rangle _t=F_S\langle \delta S\rangle +F_I\langle \delta I\rangle +F_R\langle \delta R\rangle \nonumber \\ & \quad\quad\quad +\left( 1/2\right) F_{SS}\langle \delta S^2\rangle +\left( 1/2\right) F_{II}\langle \delta I^2\rangle \nonumber \\ & \quad\quad\quad +F_{SI}\langle \delta S\delta I\rangle -k^2D_1\langle \delta S\rangle , \end{aligned}$$

and

22 $$\begin{aligned} & \langle \delta I\rangle _t=G_S\langle \delta S\rangle +G_I\langle \delta I\rangle +G_R\langle \delta R\rangle \nonumber \\ & \quad\quad\quad +\left( 1/2\right) G_{SS}\langle \delta S^2\rangle +\left( 1/2\right) G_{II}\langle \delta I^2\rangle \nonumber \\ & \quad\quad\quad +G_{SI}\langle \delta S\delta I\rangle -k^2D_2\langle \delta I\rangle . \end{aligned}$$

23 $$\begin{aligned} & \langle \delta R\rangle _t=H_I\langle \delta I\rangle +H_R\langle \delta R\rangle -k^2D_3\langle \delta R\rangle . \end{aligned}$$

As expected for a nonlinear stochastic system, we observe that Eqs. (21), and (22) involve the higher order moments - specifically $\left\langle \delta S^2\right\rangle$, $\left\langle \delta I^2\right\rangle$, and $\left\langle \delta S\delta I\right\rangle$. It follows, therefore, that the solution of Eqs. (21), (22) and (23) requires information about the evolution of these higher moments. We find the equations of motion for the higher moments using Eqs. (14), and (15). For instance, to obtain the evolution equation of $\left\langle \delta S^2\right\rangle$, we multiply Eq. (14) by $2\delta S$ followed by statistical averaging. Moreover, we truncate the moments at the second order in order to break the hierarchy and achieve moment closure. Finally, in order to compute terms such as $\langle \xi \delta S\rangle$, we invoke Novikov’s theorem for Gaussian noise processes (Gardiner 2009; Bressloff 2014; Van Kampen 1992), which may be stated as

24 $$\begin{aligned} \langle f(u)\xi \rangle =C_I\langle f(u)f(u')\rangle . \end{aligned}$$

Carrying out the aforementioned procedure, we now substitute the values of the partial derivatives in Eqs. (21)–(23) to obtain:

25 $$\begin{aligned} & \langle \delta S\rangle _t=-\left[ k^2D_1+d+\frac{\beta _0 I_0^2}{1+\alpha I^2_0}\right] \langle \delta S\rangle \nonumber \\ & \quad\quad\quad -\frac{2\beta _0 S_0 I_0}{(1+\alpha I^2_0)^2}\langle \delta I\rangle +\mu \langle \delta R\rangle \nonumber \\ & \quad\quad\quad -\frac{\beta _0S_0(1-3\alpha I_0^2)}{(1+\alpha I^2_0)^3}\langle \delta I^2\rangle -\frac{2\beta _0 I_0 }{(1+\alpha I^2_0)^2}\langle \delta S\delta I\rangle . \end{aligned}$$

26 $$\begin{aligned} & \langle \delta I\rangle _t=\frac{\beta _0 I_0^2}{(1+\alpha I^2_0)}\langle \delta S\rangle \nonumber \\ & \quad\quad\quad -\left[ k^2D_2+\gamma +d-\frac{2\beta _0 S_0 I_0}{(1+\alpha I^2_0)^2}\right] \langle \delta I\rangle \nonumber \\ & \quad\quad\quad +\frac{\beta _0S_0(1-3\alpha I_0^2)}{(1+\alpha I^2_0)^3}\langle \delta I^2\rangle +\frac{2\beta _0 I_0 }{(1+\alpha I^2_0)^2}\langle \delta S\delta I\rangle . \end{aligned}$$

27 $$\begin{aligned} & \langle \delta R\rangle _t=\gamma \langle \delta I\rangle -(\mu +d+k^2D_3)\langle \delta R\rangle . \end{aligned}$$

The higher-order moments evolve as:

28 $$\begin{aligned} & \langle \delta S^2\rangle _t=-2\left[ k^2D_1+d+\frac{\beta _0 I_0^2}{1+\alpha I^2_0}\right] \langle \delta S^2\rangle \nonumber \\ & \quad\quad\quad\quad -\frac{4\beta _0 S_0 I_0}{(1+\alpha I^2_0)^2}\langle \delta S\delta I\rangle +2\mu \langle \delta S\delta R\rangle . \end{aligned}$$

29 $$\begin{aligned} & \langle \delta I^2\rangle _t=2C_I\langle \delta I\rangle -2\left[ k^2D_2+\gamma +d-\frac{2\beta _0 S_0 I_0}{(1+\alpha I^2_0)^2}\right] \langle \delta I^2\rangle \nonumber \\ & \quad\quad\quad\quad +\frac{2\beta _0 I_0^2}{(1+\alpha I^2_0)}\langle \delta S\delta I\rangle . \end{aligned}$$

30 $$\begin{aligned} & \langle \delta R^2\rangle _t=2\gamma \langle \delta I\rangle -2(\mu +d+k^2D_3)\langle \delta R^2\rangle . \end{aligned}$$

31 $$\begin{aligned} & \langle \delta S\delta I\rangle _t=C_I\langle \delta S\rangle +\frac{\beta _0 I_0^2}{(1+\alpha I^2_0)}\langle \delta S^2\rangle -\frac{2\beta _0 S_0 I_0}{(1+\alpha I^2_0)^2}\langle \delta I^2\rangle \nonumber \\ & \quad\quad\quad\quad +h_1\langle \delta S\delta I\rangle +\mu \langle \delta I\delta R\rangle , \end{aligned}$$

where $h_1$ will be subsequently defined as an element of the matrix *A* presented in Eq. (35).

32 $$\begin{aligned} & \langle \delta S\delta R\rangle _t=\mu \langle \delta R^2\rangle +\gamma \langle \delta S\delta I\rangle \nonumber \\ & \quad\quad\quad\quad\quad -\left[ k^2(D_1+D_3)+\mu +2d+\frac{\beta _0 I_0^2}{1+\alpha I^2_0}\right] \langle \delta S\delta R\rangle \nonumber \\ & \quad\quad\quad\quad\quad -\frac{2\beta _0 S_0 I_0}{(1+\alpha I^2_0)^2}\langle \delta I\delta R\rangle , \end{aligned}$$

33 $$\begin{aligned} & \langle \delta I\delta R\rangle _t=C_I\langle \delta R\rangle +\gamma \langle \delta I^2\rangle \nonumber \\ & \quad +\frac{\beta _0 I_0^2}{(1+\alpha I^2_0)}\langle \delta S\delta R\rangle \nonumber \\ & \quad -\left( k^2(D_2+D_3)\frac{}{}+\gamma +\mu +2d{}\right. \nonumber \\ & \quad \left. {}-\frac{2\beta _0 S_0 I_0}{(1+\alpha I^2_0)^2}\right) \langle \delta I\delta R\rangle . \end{aligned}$$

Next the coupled linear ordinary differential equations derived for the moments are collected in matrix form as:

34 $$\begin{aligned} \dot{X}=AX, \end{aligned}$$

where $X=(x_1, x_2, x_3, x_4, x_5,x_6,x_7,x_8,x_9)^T$, and *A* is a $9\times 9$ matrix. The components $x_i$ are given as $x_1=\langle \delta S\rangle$, $x_2=\langle \delta I\rangle$, $x_3=\langle \delta R\rangle$, $x_4=\langle \delta S^2\rangle$, $x_5=\langle \delta I^2\rangle$, $x_6=\langle \delta R^2\rangle$, $x_7=\langle \delta S\delta I\rangle$, $x_8=\langle \delta S\delta R\rangle$, $x_9=\langle \delta I\delta R\rangle$. The matrix *A* is obtained as

35 $$\begin{aligned} A=\begin{bmatrix} a_1& a_2& a_3& 0& a_5& 0& a_7& 0& 0\\ b_1& b_2& 0& 0& b_5& 0& b_7& 0& 0\\ 0& c_2& c_3& 0& 0& 0& 0& 0& 0\\ 0& 0& 0& 2a_1& 0& 0& 2a_2& 2a_3& 0\\ 0& 2C_I& 0& 0& 2b_2& 0& 2b_1& 0& 0\\ 0& 0& 0& 0& 0& 2c_3& 0& 0& 2c_2\\ C_I& 0& 0& b_1& a_2& 0& h_1& 0& a_3\\ 0& 0& 0& 0& 0& a_3& c_2& h_2& a_2\\ 0& 0& C_I& 0& c_2& 0& 0& b_1& h_3 \end{bmatrix}, \end{aligned}$$

where $h_1=a_1+b_2,~h_2=a_1+c_3,~h_3=b_2+c_3$, and other matrix elements are defined as:

36 $$\begin{aligned} \begin{aligned} a_1&=-\left[ k^2D_1+d+\frac{\beta _0 I_0^2}{1+\alpha I^2_0}\right] ,a_2=-\frac{2\beta _0 S_0 I_0}{(1+\alpha I^2_0)^2}\\ a_3&=\mu ,a_5=-\frac{\beta _0S_0(1-3\alpha I_0^2)}{(1+\alpha I^2_0)^3},\\ a_7&=-\frac{2\beta _0 I_0}{(1+\alpha I^2_0)^2},~b_1=\frac{\beta _0 I_0^2}{(1+\alpha I^2_0)},\\ b_2&=-(k^2D_2+\gamma +d+a_2),~b_5=-a_5,~b_7=-a_7,\\ c_2&=\gamma , ~c_3=-(\mu +d+k^2D_3). \end{aligned} \end{aligned}$$

Recalling our focus on analyzing the stability characteristics and pattern formation with respect to $\alpha$ (the saturation parameter), $\mu$ (the re-infection parameter), $D_1$ (the diffusion coefficient of *S*) and $C_I$ (the noise intensity), we now present the results in the next section.

## Results

The results broadly fall under the two categories of those pertaining to stability analysis and self-organized pattern formation, and we present them in that sequence.

**Fig. 1:**
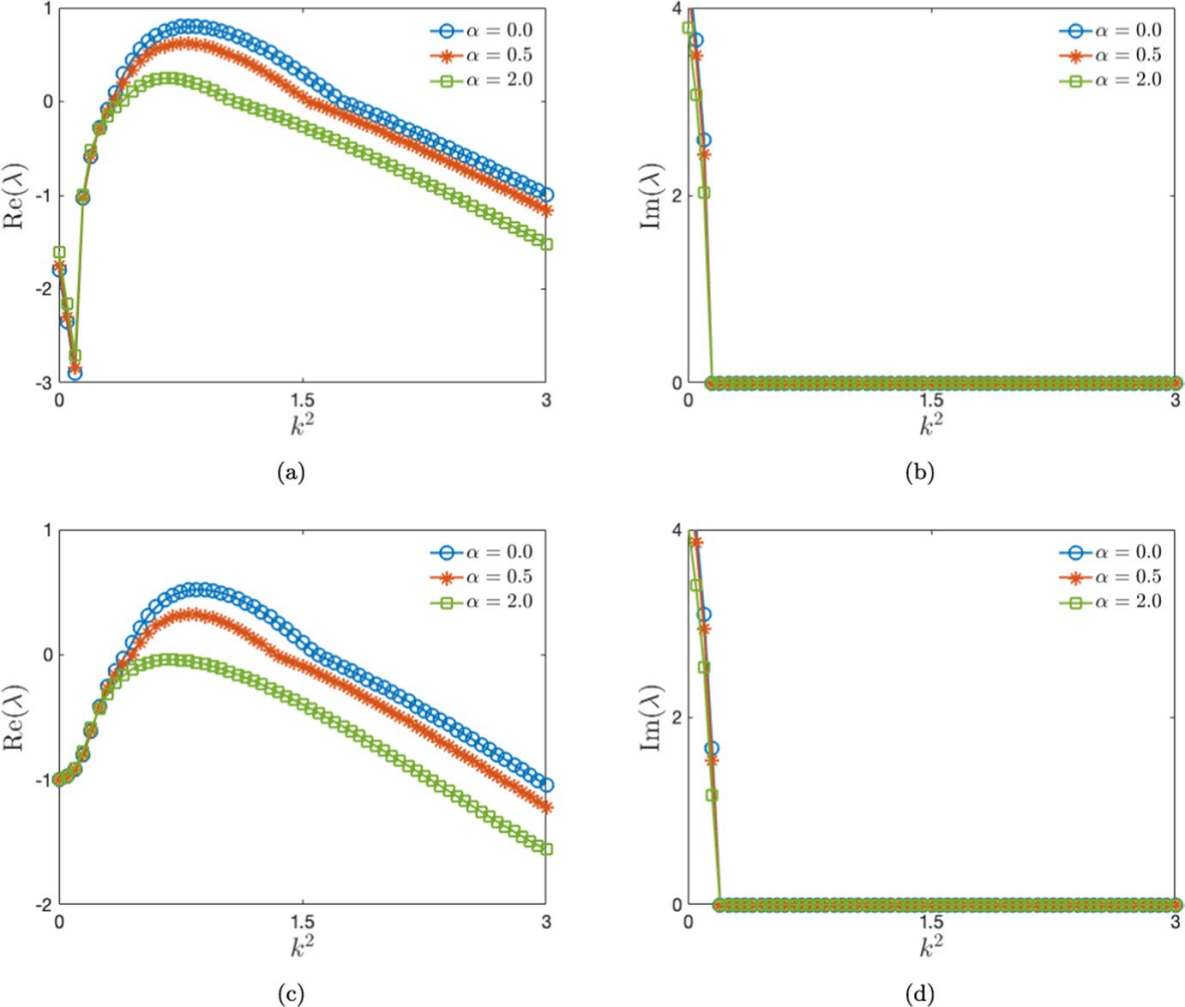
The variation of real (left panel) and imaginary (right panel) parts of eigenvalue $\lambda$ with the square of wave number is shown. We take (**a**, **b**) $\mu =0.0$, (**c**, **d**) $\mu =0.1$. The value of $D_1=10.0$

For the stability analysis, we plot the real and imaginary parts of the maximal eigenvalue with the square of wave number ($k^2$) in Fig. 1. The range of k-values shrinks as we increase the saturation parameter. This behavior persists even in the presence of re-infection ($\mu =0.1$). Furthermore, from the Fig. 1a, we see that the threshold k-values are distinct for different $\alpha -$values, for instance, at $\mu =0.0$, $\alpha =0.0$ corresponds to threshold $k=0.57$, $\alpha =0.5$ corresponds to threshold $k=0.58$ and $\alpha =2.0$ corresponds to threshold $k=0.62$. We observe a similar trend in Fig. 1c as well. Moreover, the peak values of the maximal eigenvalue for increasing $\alpha -$values also shift to the region of lower *k* values.

**Fig. 2:**
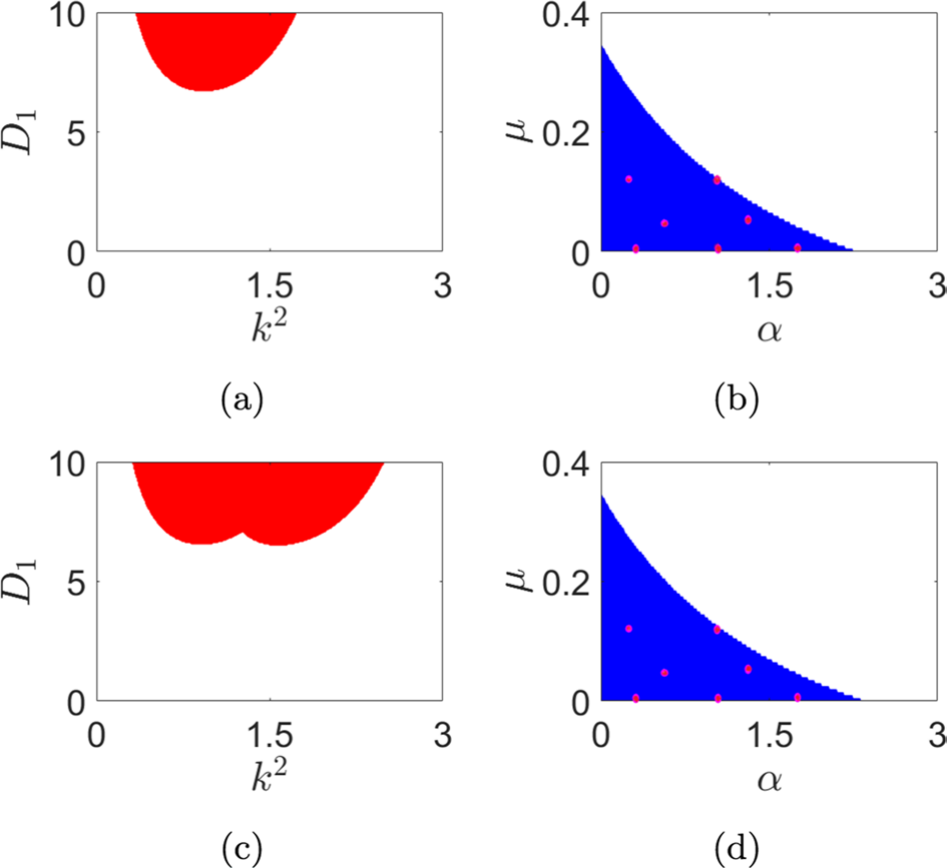
Region of instabilities is shown in $k^2-D_1$ plane on left panel and $\alpha -\mu$ plane on right panel. We take (**a**, **b**) $C_I=0.0$, (**c**, **d**) $C_I=8.0\times 10^{-2}$. For the left panel: $\alpha =0.0, \mu =0.0$. For the right panel, $D_1=10.0$, however, we can choose any value from (a) in the shaded region

For stability analysis in parameter space, we solve for the maximal eigenvalue ($\lambda$) of the matrix *A* of Eq. (35) for each case obtained by varying parameters of interest. Points (or regions) in parameter space for which one obtains positive maximal eigenvalues indicate instability. Specifically, the threshold value $k_{th}$ of *k* corresponding to a Turing bifurcation may be obtained by simultaneously solving ($Im~\lambda (k))=0$ and ($Re~\lambda (k))=0$ for $k=k_{th}$. We first consider the case where the reinfection parameter $\mu$ and the saturation parameter $\alpha$ are both set to zero. For this case, in Fig. 2a, we present the plot of the maximal eigenvalue in the plane formed by the square of wave number ($k^2$) and the diffusion coefficient ($D_1$) of the susceptible population, in the *deterministic* sub-case ($C_I=0.0$). The corresponding plot for the *stochastic* sub-case with $C_I=8.0\times 10^{-2}$ is presented in Fig. 2c. The *unstable* regions in these plots, i.e. the areas corresponding to positive maximal eigenvalues, are indicated in red colour.

Turning next to the case of non-vanishing reinfection $\mu$ and saturation $\alpha$ parameters, we obtain stability plots in the $\alpha - \mu$ plane. The deterministic case ($C_I=0.0$) is presented in Fig. 2b and the stochastic case with $C_I=8.0\times 10^{-2}$ is presented in Fig. 2d. Note that while we have chosen $D_1=10.0$ in obtaining these plots, any value of $D_1$ from the unstable (red-coloured) region in Fig. 2a will yield similar results. Finally, we note the numerical values of the other system parameters that are held identical across all the aforementioned cases while obtaining stability plots. These are: $b=1.0$, $d=1.0$, $\beta _0=35.0$, $\gamma =1.5$, $D_2=1.0$ and $D_3=0.2$.

We observe from Fig. 2a that, in the absence of spatial diffusion ($D_1=0.0$) and noise ($C_I=0.0$), the homogeneous stationary state $(S_0,I_0,R_0)$ is stable for the previously indicated set of other parameter values. However, the plot in Fig. 2a also indicates fundamental changes in stability characteristics in the presence of diffusion - instabilities in the homogeneous stationary state emerge for a range of values of the diffusion constant $D_1$. More specifically, under noise-free conditions, we observe unique *diffusion-driven* (i.e. Turing-type) instabilities arising for $D_1$ values lying in the shaded region in the $k^2-D_1$ plane Fig. 2a. Notably, the largest eigenvalue is positive in the shaded region over a finite range of $k^2$. This range is determined by the other system parameters in the deterministic case and also by the noise intensity in the stochastic case.

**Fig. 3:**
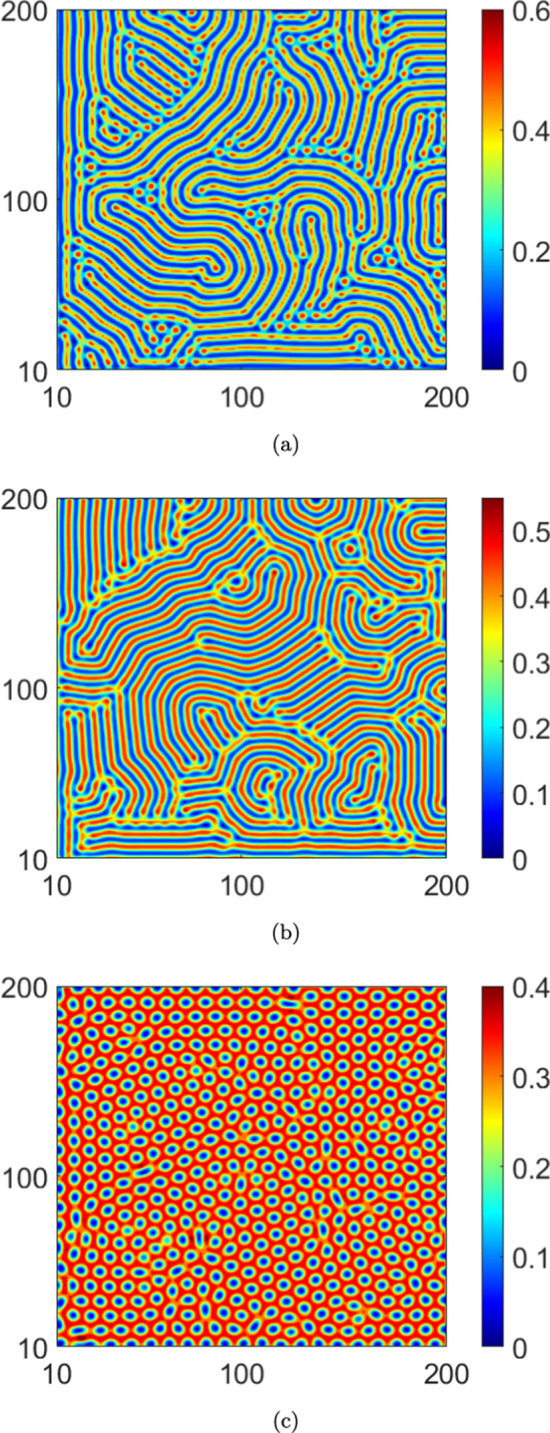
The pattern of infected population density *I* in $x-y$ plane for deterministic case ($C_I=0.0$) and $\mu =0.0$: (**a**) $\alpha =0.0$, (**b**) $\alpha =0.5$ and (**c**) $\alpha =2.0$. We take $D_1=10.0$

**Fig. 4:**
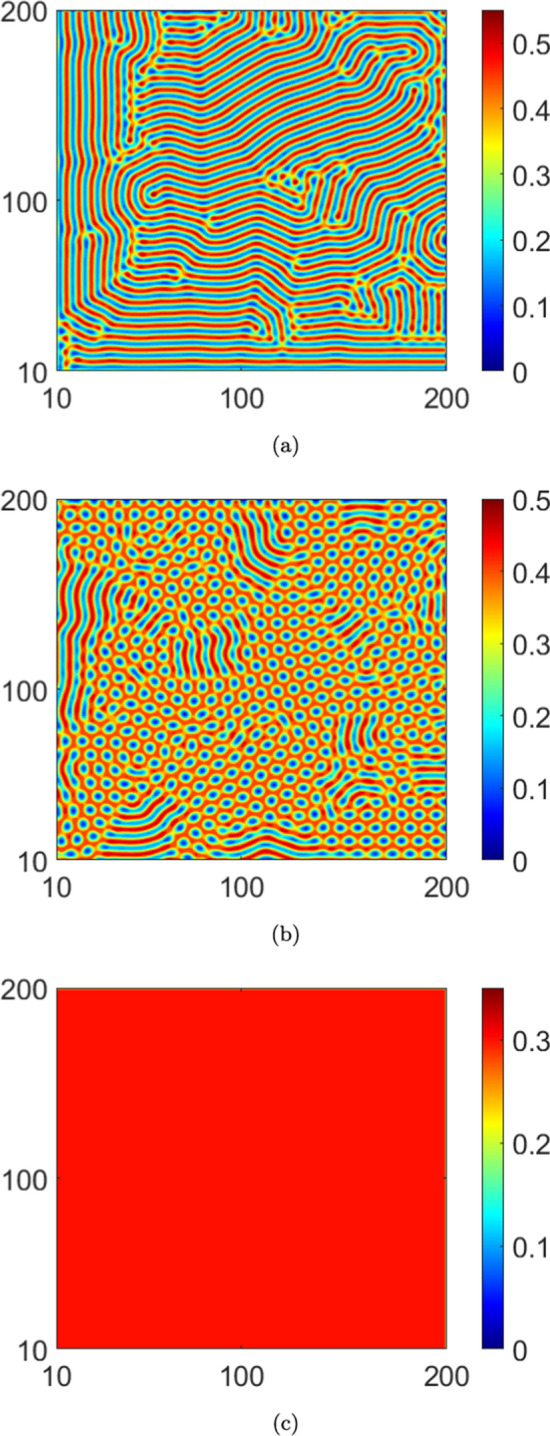
The pattern of infected population density *I* in $x-y$ plane for deterministic case ($C_I=0.0$) and $\mu =0.1$. (**a**) $\alpha =0.0$, (**b**) $\alpha =0.5$ and (**c**) $\alpha =2.0$. We take $D_1=10.0$

**Fig. 5:**
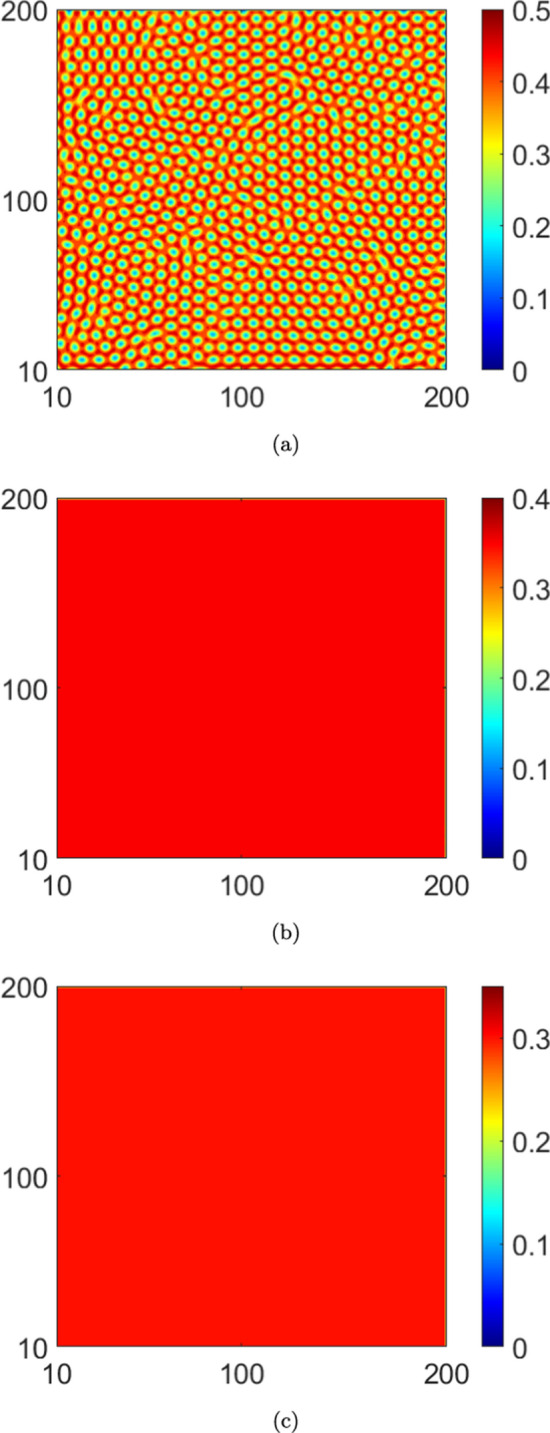
The pattern of infected population density *I* in $x-y$ plane for deterministic case ($C_I=0.0$) and $\mu =0.3$. (**a**) $\alpha =0.0$, (**b**) $\alpha =0.5$ and (**c**) $\alpha =2.0$. We take $D_1=10.0$

**Fig. 6:**
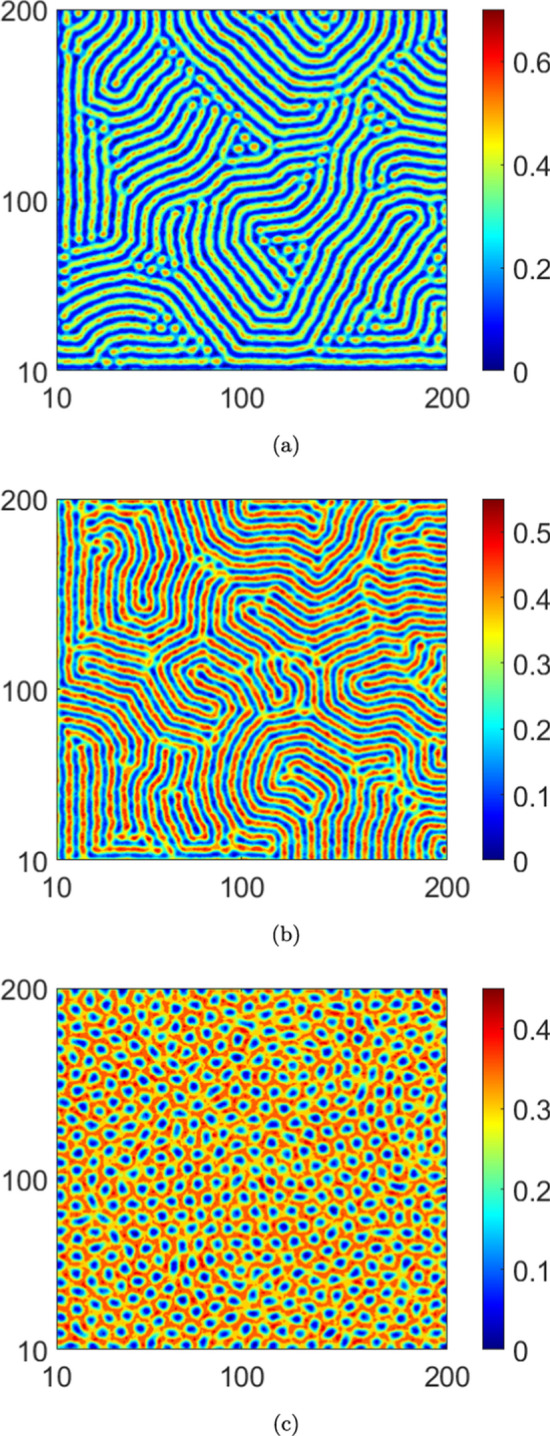
The pattern of infected population density *I* in $x-y$ plane for noisy case ($C_I=8\times 10^{-2}$) and $\mu =0.0$. (**a**) $\alpha =0.0$, (**b**) $\alpha =0.5$ and (**c**) $\alpha =2.0$. We take $D_1=10.0$

**Fig. 7:**
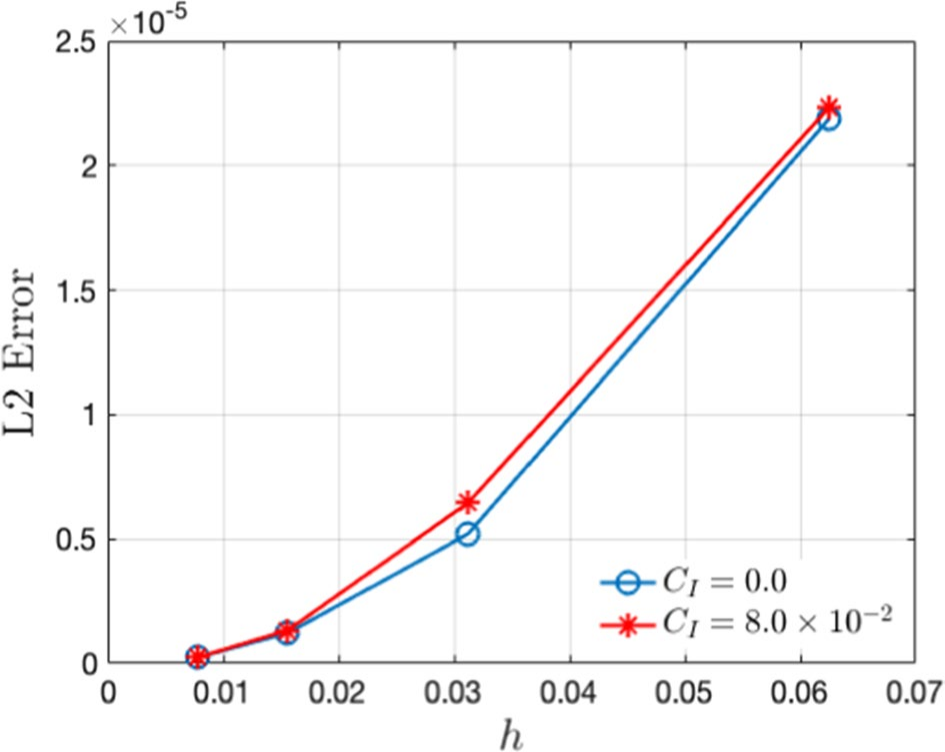
The convergence of the simulation is obtained by plotting $L_2$ error with grid spacing *h* in two distinct cases, i.e., without noise ($C_I=0.0$) and with noise ($C_I=8\times 10^{-2}$)

The instabilities described in the previous paragraphs lead to self-organized, stationary spatial patterns in equilibrium. Choosing system parameters guided by the stability results, we investigate next, pattern formation in the stationary infected population density *I*(*x*, *y*). Numerically solving for the stationary solutions of the PDEs Eq. (2), we present contour plots of *I*(*x*, *y*) in Figs. 3–10. To obtain the numerical solutions, we use a central difference scheme over a grid size of $200\times 200$, with spatial step sizes $\Delta x=\Delta y=1.0$. Note that the grid size is chosen in order to accommodate a smaller wave number as well, over which the largest eigenvalue remains positive. Thus, to harbour larger wavelengths (corresponding to smaller *k*), a relatively large domain size is essential. The numerical scheme runs for a time step of $\Delta t=0.01$, while imposing no flux boundary conditions on the boundaries. We then plot the equilibrium infected population density *I*(*x*, *y*) on the $x-y$ plane in a range of (10, 200) on each axis. The system parameters held identical across all cases in the numerical solutions are: $b=1.0$, $d=1.0$, $\beta _0=35.0$, $\gamma =1.5$, $D_2=1.0$ and $D_3=0.2$. Distinct patterns are obtained by varying $\alpha$, $\mu$, $D_1$ and $C_I$ sequentially, numerical values of which are provided in the corresponding figure captions.

The spatial patterns in the case with no reinfections ($\mu =0.0$) are presented in Fig. 3 wherein we observe a spot pattern for a higher saturation level ($\alpha =2.0$). Moreover, we note depletion in infected population density with increasing saturation levels. Next, the patterns obtained by taking reinfection into account are presented in Figs. 4 and 7 for $\mu =0.1$ and $\mu =0.3$, respectively. In particular, in Fig. 4, no patterns are observed in the case of higher saturation ($\alpha =2.0$). Indeed, this is consistent with our stability results since we do not see any instability in Fig. 2b for this $\alpha$ value with $\mu =0.1$. Moreover, if we were to increase the reinfection parameter to $\mu =0.3$, from Fig. 2b, we see that instabilities do not emerge for higher values of the saturation parameter, i.e., $\alpha =0.5$ and $\alpha =2.0$. Therefore, no pattern formation occurs for these $\alpha$- values. A comparison of the plots in Figs. 4 and 7 with those in Fig. 3 indicates that an increase in reinfections promotes the stability of the equilibrium solution. This is also true for an increase in saturation levels. However, increasing the saturation levels reduces the value of the infected population density in each figure.

Next, we analyze the effects of noise on pattern formation, both with and without reinfection. We present the patterns corresponding to the deterministic case in Fig. 3 and those corresponding to the stochastic case in Fig. 10. Interestingly, a comparison of the plots in Fig. 3 and Fig. 10 indicates that the infected population density is higher in equilibrium in the absence of noise. For a convergence check of the simulations, we plot, in logarithmic scale, the $L_2$ norm error in the infected population density (*I*(*x*, *y*)) with respect to the grid spacing ($h=dx = dy$) in Fig. 7. This is computed for both the deterministic and stochastic cases. From the data plotted in the figure, the order of convergence in the deterministic case is 2.15, while for the noisy case ($C_I=8.0\times 10^{-2}$) it is 1.83. While the order of convergence is higher in the deterministic case (as is to be expected), the numerical values obtained in both cases indicate robust convergence of the simulations (Bernkopf and Melenk 2024).

## Discussion and Conclusions

In this article, we presented a study of instabilities and the consequent emergence of self-organized Turing patterns in a nonlinear stochastic system of PDEs representing a spatiotemporal system with a closed loop of dynamic compartmental transitions. To the best of our knowledge, this is one of the first efforts in this direction. From the standpoint of a closed-loop system, our results show that both the onset of instability and the self-organization behavior are significantly altered as a consequence of the loop closure. Specifically, in the context of the epidemic model with reinfections that exemplifies such a system in our study, varying the reinfection parameter altered the pattern formation both qualitatively and quantitatively. For example, if the reinfection parameter is increased in the absence of saturation, the self-organized patterns approach the spotted structure with higher values of infected population density. On the other hand, under higher saturation levels, increasing the value of the reinfection parameter leads to stable behaviour. Thus, our results affirm that lower values of the reinfection parameter promote the formation of Turing patterns. It is interesting to consider how this correlates with a fundamental feature of Turing patterns - that they emerge only in the presence of two closely competing species. In this case, lower values of the reinfection parameter ensure that the infected population density would not overwhelmingly dominate the susceptible population density but rather that the two would be engaged in close competition.

We now turn to further discussing the results from the viewpoint of epidemic dynamics. Firstly, our results highlight the pivotal role played by higher-order analysis in uncovering instabilities in epidemic spread dynamics influenced by nonlinearities (such as those arising from saturation effects), stochastic effects, or a combination thereof. It bears emphasis that linear stability analysis would be incapable of revealing such instabilities.

Secondly, the regions of instability in Fig. 2b, d affirm competing behaviour between the reinfection and saturation mechanisms. Indeed, this is intuitive since reinfection implicitly contributes to the infected population density, thereby inhibiting saturation.

Thirdly, it is of interest to consider the consequences of the competition between reinfection and saturation on the pattern formation. Our results indicate that larger saturation levels drive the system towards a spot pattern as it evolves to a stationary state from non-stationary states. However, these spot patterns interestingly emerge at lower saturation levels owing to the reinfection.

Fourth, the results indicate that the system does not exhibit instability at higher reinfection and saturation parameter values. Either of the following are plausible reasons for this behaviour: (*i*) the equilibrium solutions are complex functions, (*ii*) higher saturation levels promote a more spatially uniform infected population density with evolving time, thereby inhibiting the formation of spatially periodic patterns. Note that a similar situation prevails for higher values of the reinfection parameter as well. More broadly, higher values of either the saturation or reinfection parameters inhibit competition between the infected and susceptible population densities; we reiterate that such competition is essential for the occurrence of patterns. Fifth, the results offer insights into the important question of the effects of the interaction between noise and nonlinearity on the stability characteristics and pattern formation. We note, from the stability diagrams Fig. 2a, c that, in the absence of both reinfection and saturation, the influence of noise expands the region of instability, with respect to the wave number *k*. In other words, in the presence of noise, the range of values of the diffusion coefficient and the wave number that support the onset of instabilities become extended. Furthermore, the results also indicate that the competing behaviour between the saturation and the reinfection mechanisms tends to be robust to noisy perturbations. Finally, the patterns that emerge in the stochastic cases stand in sharp contrast to those obtained in the corresponding deterministic cases. Specifically, the patterns show that lower magnitudes of the infected population density are realized in the stochastic cases compared to the corresponding deterministic cases. Moreover, the spot patterns are also distinct in the two cases (see Figs. 3 and 6c).

The results suggest several potential directions for further research. While the present effort focused on the effects of loop closure on the stability characteristics and self-organised patterns in the context of an epidemic model, the higher-order stability analysis discussed here is expected to provide insights into problems such as prey-predator dynamics where similar loop closure plays a crucial role (Brown et al. 2004; Tirok et al. 2011; Fryxell et al. 2019). In epidemic models, the stability and pattern formation in the presence of multiple pathogen variants are worthy of investigation. For instance, in the case of COVID-19, the emergence and simultaneous circulation of multiple variants – such as the $\alpha$ and $\delta$ variants of SARS-COV-2 (Zhan et al. 2023; Liossi et al. 2023) – was a critical factor that determined the overall trajectory of the epidemic. Finally, the *controlled* realisation of patterns would be yet another interesting direction for further research. While feedback control of the Turing instability has been discussed in the literature (for instance, in the context of the Ginzsburg-Landau equation (Golovin et al. 2009)), applying control–theoretic methods informed by the results of this article to achieve improved mitigation (where the control would correspond to interventions imposed by public health administrations such as social distancing or masking) would be of interest. We conclude with the hope that the results reported in this article motivate further research on instabilities and self-organized pattern formation in spatiotemporal nonlinear stochastic dynamical systems and their applications.

## Appendix

The deterministic SIR model (Ruan and Wang 2003)

A1 $$\begin{aligned} \frac{\partial }{\partial \tau }s(x,y,t)&=b'n-d's-\frac{1}{n}\frac{\beta s i^2}{1+\alpha _0 i^2}+\mu ' r+d_s\nabla '^2 s,\end{aligned}$$

A2 $$\begin{aligned} \frac{\partial }{\partial \tau }i(x,y,t)&=\frac{1}{n}\frac{\beta s i^2}{1+\alpha _0 i^2}-(\gamma '+d') i+d_i\nabla '^2 i,\end{aligned}$$

A3 $$\begin{aligned} \frac{\partial }{\partial \tau } r(x,y,t)&=\gamma ' i-(\mu '+d') r+d_r\nabla '^2r, \end{aligned}$$

where *s*, *i* and *r* denote the densities of susceptible, infected and recovered population respectively and $n=s(x,y,t)+i(x,y,t)+r(x,y,t)$. The the LHS of Eq. (A.1) is in the unit of number of individual (susceptible, infected or recovered) per unit time, $b'n$ is the birth rate of *s* population, $d'$ is the death rate, of *s*, *i* and *r*, $\beta$ is the rate of infection per capita per unit area, $\alpha _0$ is the saturation parameter in the unit of inverse of population density, $\mu '$ is the rate of re-infection of *r*, $\gamma '$ is the recovery rate of *i*, $d_s,~d_i$ and $d_r$ are diffusivities of *s*, *i* and *r* respectively in the unit of $\frac{[Length]^2}{[Time]}$. The Laplacian operator $\nabla '^2=\frac{\partial ^2}{\partial x^2}+\frac{\partial ^2}{\partial y^2}$ is in the unit of $[Lenth]^{-2}$. We non-dimensionalize the system (A.1) by following substitution as: $S=s/n,~I=i/n,~R=r/n$, $t=\gamma ' \tau ,~ X=\sqrt{\gamma '} x/\sqrt{d_i}$, and $Y=\sqrt{\gamma '}y/\sqrt{d_i}$. We now consider the following system of coupled PDEs for *S*, *I* and *R* (Ruan and Wang 2003):

A4 $$\begin{aligned} \frac{\partial S}{\partial t}&=b-dS-\frac{\beta _0 S I^2}{1+\alpha I^2}+\mu R+D_1\nabla ^2 S, \end{aligned}$$

A5 $$\begin{aligned} \frac{\partial I}{\partial t}&=\frac{\beta _0S I^2}{1+\alpha I^2}-(\gamma +d) I+D_2\nabla ^2I+\xi \left( t,x,y\right) , \end{aligned}$$

A6 $$\begin{aligned} \frac{\partial R}{\partial t}&=\gamma I-(\mu +d) R+D_3\nabla ^2R \end{aligned}$$

$b=b'/\gamma ', d=d'\gamma ', \beta _0=\beta n/\gamma ', \alpha =\alpha _0 n/\gamma ', \mu =\mu '/\gamma '$, $D_1=d_s/d_i, D_2=d_i/d_i=1,$ and $D_3=d_r/d_i$. We retain the symbol $D_2$ and later in the results section we have assigned it a value as unity.
