# Correction: The Emergence of Turing Instability and Pattern Formation in a Nonlinear Stochastic Spatiotemporal Epidemic Model with Reinfections

**DOI:** 10.1007/s10441-026-09528-5

**Published:** 2026-05-23

**Authors:** Aman Kumar Singh, Rasha Almshekhs, Manish Kumar, Subramanian Ramakrishnan

**Affiliations:** 1 Department of Physics, Maharshi Vishwamitra College, Buxar, BR 802101 India; 2 Department of Mechanical and Aerospace Engineering, University of Dayton, Dayton, OH 45469 USA; 3 Department of Mechanical and Materials Engineering, University of Cincinnati, Cincinnati, OH 45221 USA

**Correction to: Acta Biotheoretica (2026) 74:10**

https://doi.org/10.1007/s10441-026-09518-7

In this article, the author’s name Rasha Almshekhs was incorrectly written as Rasha Almsheks.

The original article has been corrected.

